# Cardiovascular Disease and Other Competing Causes of Death in Older Kidney Cancer Patients

**DOI:** 10.31083/RCM25277

**Published:** 2025-01-14

**Authors:** Yinglan Liang, Liangjia Zeng, Ruoyun Zhou, Manting Feng, Linglong Liu, Kexin Chen, Jinqi Huang, Haowen Liang, Baixin He, Binghua Zhang, Yican Ying, Yuerong Chen, Tianwang Guan, Min Yi

**Affiliations:** ^1^Department of Anesthesiology, The Second Clinical College of Guangzhou Medical University, 510180 Guangzhou, Guangdong, China; ^2^Cardiovascular Medicine and Cardio-Oncology Group, Medical Exploration and Translation Team, 510000 Guangzhou, Guangdong, China; ^3^Department of Clinical Medicine, The Nanshan Clinical College of Guangzhou Medical University, 510180 Guangzhou, Guangdong, China; ^4^Department of Clinical Medicine, The Third Clinical College of Guangzhou Medical University, 510180 Guangzhou, Guangdong, China; ^5^Department of Clinical Medicine, The Second Clinical College of Guangzhou Medical University, 510180 Guangzhou, Guangdong, China; ^6^Department of Clinical Medicine, The First Clinical College of Guangzhou Medical University, 510180 Guangzhou, Guangdong, China; ^7^Department of Clinical Medicine, The Sixth Clinical College of Guangzhou Medical University, 510180 Guangzhou, Guangdong, China; ^8^Minimally Invasive Tumor Therapies Center, Guangdong Second Provincial General Hospital, 510317 Guangzhou, Guangdong, China; ^9^Guangdong Engineering Research Center of Boron Neutron Therapy and Application in Malignant Tumors, Dongguan Key Laboratory of Precision Diagnosis and Treatment for Tumors, Dongguan Engineering Research Center for Innovative Boron Drugs and Novel Radioimmune Drugs, Cancer Center, the 10th Affiliated Hospital of Southern Medical University, Southern Medical University, Guangzhou 510280, China; ^10^Department of Endocrinology, The Second Affiliated Hospital of Guangzhou Medical University, 510260 Guangzhou, Guangdong, China

**Keywords:** kidney cancer, older patients, cause of death, cardiovascular disease death, cardio-oncology

## Abstract

**Background:**

To study the risk of cardiovascular disease (CVD) and other competing causes of death in older kidney cancer patients.

**Methods:**

Data on older patients (aged 65 and above) diagnosed with kidney cancer between 1975 and 2018 were extracted from the Surveillance, Epidemiology, and End Results (SEER) database. We delved into the distribution of CVD and other competing causes of death across the entire cohort and in various patient subgroups. The competing risk analysis was used to produce cumulative mortality curves based on cumulative mortality for the primary outcomes by follow-up period. Utilizing the standardized mortality ratios (SMRs) and absolute excess risks (AERs), we contrasted the risk of CVD and other competing causes of death in older kidney cancer patients to that observed in the general population.

**Results:**

The analysis included 29,349 older kidney cancer patients, of which included 4563 CVD deaths. As survival time extended, the proportion of non-cancer deaths increased in older kidney cancer patients, with CVD accounting for the largest share of non-cancer deaths. At 10–15 years after diagnosis, cumulative non-cancer mortality exceeded primary kidney cancer as the predominant cause of death, and cumulative CVD mortality is higher among all non-cancer causes. Older kidney cancer patients exhibited a greater risk of CVD and other non-cancer deaths than their counterparts in the general older population did (SMR: 1.38–2.81; AER: 1.1–143.69).

**Conclusions:**

As survival time increases, the risk of non-cancer death in older kidney cancer patients gradually surpassed that of primary cancer, and CVD death accounted for the majority of non-cancer deaths. Among older kidney cancer patients, the risk of CVD mortality was higher than in the general population. Managing non-cancer deaths, especially CVD deaths, should be a focus in the care of older kidney cancer patients.

## 1. Introduction

Kidney cancer ranks as the 14th most prevalent cancer globally [1], with 434,419 new cases and 155,702 deaths reported worldwide in 2022 [2]. The incidence of kidney cancer is estimated to rise by 1.5% annually, indicating a sustained upward trend [3]. Kidney cancer is common in older adults, with individuals over 65 years old constituting 70% of new cases annually [4], and the peak incidence occurring at 75 years old [5]. The aging population forecasts a heightened burden on older kidney cancer patients [6, 7]. Understanding the causes of death in older kidney cancer patients is pivotal for enhancing prognostic strategies.

The burgeoning field of cardio-oncology has found that non-cancer causes, particularly cardiovascular disease (CVD), are significant contributors to mortality among cancer survivors [8, 9, 10]. Frailty becomes progressively more prevalent in the elderly as they age and makes them more susceptible to comorbid CVD [11]. The cardiovascular toxicity associated with anticancer treatments and the biology of the cancer also contribute to the wide-ranging multisystem effects [12, 13, 14]. Kidney cancer and CVD have shared risk factors, which include both behavioral characteristics (e.g., tobacco) as well as metabolic factors (e.g., obesity, hypertension) [15, 16]. Multiple reasons contribute to the complexity of older kidney cancer patients.

The risk of CVD and other competing causes of death in older kidney cancer patients is not yet clear. Existing studies have mainly focused on cause-specific mortality in the general kidney cancer patients or in patients at specific stages of kidney cancer [12, 17, 18, 19]. However, the applicability of these findings to older kidney cancer patients is debatable due to age-related disparities and limitation of specific stages. Some investigations indicated a heightened risk of CVD among older patients with multiple cancer types (kidney and renal pelvis cancer), but due to the heterogeneity of cancer, the results of multiple types of cancer may not be applicable to patients with single kidney cancer [8, 20]. Moreover, differences in causes of death across particular subgroups of older kidney cancer patients remains unexplored. Therefore, there is an urgent imperative for further research to delineate the risk associated with CVD and other competing causes of death in older kidney cancer patients.

To address this gap, we undertook a population-based analysis to determine the risk of CVD and other competing causes of death in older kidney cancer patients, and juxtaposed their risk of CVD and other competing causes of death against that of the general population. These insights furnish a scientific foundation for enhancing prognostic approaches and tailored management strategies for older kidney cancer patients.

## 2. Materials and Methods

### 2.1 Data Source

For this study, data were sourced from the Surveillance, Epidemiology, and End Results (SEER) Program (http://www.seer.cancer.gov), which is a publicly available, federally sponsored database containing data from 18 cancer registries across the United States (US), encompassing around 48% of the national population [21]. The data of multiple causes of death in US general population as standard cohort was downloaded from the Centers for Disease Control and Prevention Wide-ranging Online Data for Epidemiologic Research (CDC WONDER) [22].

### 2.2 Study Population

We extracted data of patients meeting these inclusion criteria as follows: (1) diagnosed with kidney cancer as the primary cancer; (2) diagnosed between 1975 and 2018; (3) active follow-up and clear cause of death; (4) without multiple primary cancers and (5) not diagnosed by autopsy or death certificate. The exclusion criteria included: (1) age at diagnosis <65 and (2) unknown race. The data processing is presented in **Supplementary Fig. 1**.

### 2.3 Outcome and Variables

The primary outcome of interest was death from any cause among older kidney cancer patients. The causes of death were identified using death certificates and verified by the attending physician. In the SEER database, all causes of death were classified according to International Classification of Diseases, 10th Revision (ICD-10) codes and recorded by the National Cancer for Health Statistics [23]. Detailed information for ICD-10 codes of causes of death used in this study was available in **Supplementary Table 1**. The follow-up period spanned from the initial kidney cancer diagnosis to either the date of death or the last follow-up on December 31, 2018. Patients who were alive at the final of the follow-up period were treated as censored observations. The variables are as follows: sex (male and female); race (White, Black and other); SEER stage (localized, regional, distant and unknown); grade (low, high, other and unknown) [24]; year of diagnosis (1975–1983, 1984–1993, 1994–2003, 2004–2018) [19]; survival time (<1, 1–3, 3–5, 5–10, 10–15, 15+ years), surgery (yes, no and unknown), radiotherapy (No/unknown, Yes) and chemotherapy (No/unknown, Yes). The missing data were included as category “Unknown” in variables.

### 2.4 Study Design and Statistical Analysis

We first explored the proportion of death in overall cohort by survival time and subgroups (divided by sex, race, year of diagnosis, SEER stage, grade, surgery, radiotherapy and chemotherapy). As previously reported, the proportion of deaths due to specific causes was determined by dividing the number of deaths from each cause by the total number of deaths [25]. Next, the competing risk analysis was used to produce cumulative mortality curves based on the cumulative mortality for the primary outcomes by follow-up period. Last, we computed the standardized mortality ratios (SMRs) and absolute excess risks (AERs) of CVD and other competing causes of death by survival time in older kidney cancer patients, supplemented by the SMRs and AERs of CVD by subgroups, aiming to represent the relative risk of death among older kidney cancer patients in comparison to the general older population in the US. SMR was a ratio derived by dividing observed deaths by expected deaths [26]. Additionally, AERs were calculated by using the formula: AERs = 10,000 (observed deaths – expected deaths)/(person-years at risk) [27]. All computations were performed using R software (version 4.1.3, R Foundation for Statistical Computing, Vienna, Austria), with statistical significance set at a *p* value < 0.05.

## 3. Results

### 3.1 Participant Characteristics

In total, 29,349 older kidney cancer patients who were diagnosed between 1975 and 2018 were included, of whom 59.5% were male and 85.7% were white (Table 1). Among them, 9735 patients died from primary cancer, while 8854 patients died from non-cancer causes, including 4563 CVD deaths. Over half of the patients (52.4%) and 93.1% of survivors were diagnosed between 2004 and 2018. The majority of patients had localized (53.9%) or regional (21.7%) tumors; 32.6% had low-grade tumors; most (81.6%) underwent surgery, while the frequencies of chemotherapy (7.3%) and radiotherapy (7.7%) were relatively low.

**Table 1.** **Characteristics of included older kidney cancer patients**.

		Alive	Primary cancer	Other cancer	CVD	Other noncancer diseases	Overall	*p*
		(N = 9602)	(N = 9735)	(N = 1158)	(N = 4563)	(N = 4291)	(N = 29,349)	
Gender								<0.001
	Male	5672 (59.1%)	5956 (61.2%)	726 (62.7%)	2638 (57.8%)	2485 (57.9%)	17,477 (59.5%)	
	Female	3930 (40.9%)	3779 (38.8%)	432 (37.3%)	1925 (42.2%)	1806 (42.1%)	11,872 (40.5%)	
Race								<0.001
	White	7886 (82.1%)	8588 (88.2%)	1022 (88.3%)	3951 (86.6%)	3697 (86.2%)	25,144 (85.7%)	
	Black	829 (8.6%)	557 (5.7%)	81 (7.0%)	385 (8.4%)	348 (8.1%)	2200 (7.5%)	
	Other^1^	887 (9.2%)	590 (6.1%)	55 (4.7%)	227 (5.0%)	246 (5.7%)	2005 (6.8%)	
Year of diagnosis								<0.001
	1975–1983	5 (0.1%)	1593 (16.4%)	181 (15.6%)	729 (16.0%)	404 (9.4%)	2912 (9.9%)	
	1984–1993	53 (0.6%)	2168 (22.3%)	278 (24.0%)	1300 (28.5%)	978 (22.8%)	4777 (16.3%)	
	1994–2003	600 (6.2%)	2489 (25.6%)	320 (27.6%)	1371 (30.0%)	1489 (34.7%)	6269 (21.4%)	
	2004–2018	8944 (93.1%)	3485 (35.8%)	379 (32.7%)	1163 (25.5%)	1420 (33.1%)	15,391 (52.4%)	
Survival time^2^								<0.001
	<1 year	1431 (14.9%)	4993 (51.3%)	567 (49.0%)	696 (15.3%)	635 (14.8%)	8322 (28.4%)	
	1–3 years	2103 (21.9%)	2409 (24.7%)	207 (17.9%)	719 (15.8%)	589 (13.7%)	6027 (20.5%)	
	3–5 years	1602 (16.7%)	1030 (10.6%)	96 (8.3%)	625 (13.7%)	559 (13.0%)	3912 (13.3%)	
	5–10 years	2534 (26.4%)	943 (9.7%)	154 (13.3%)	1246 (27.3%)	1211 (28.2%)	6088 (20.7%)	
	10–15 years	1317 (13.7%)	285 (2.9%)	87 (7.5%)	763 (16.7%)	765 (17.8%)	3217 (11.0%)	
	15 years+	615 (6.4%)	75 (0.8%)	47 (4.1%)	514 (11.3%)	532 (12.4%)	1783 (6.1%)	
SEER stage								<0.001
	Localized	7331 (76.3%)	1927 (19.8%)	339 (29.3%)	3202 (70.2%)	3030 (70.6%)	15,829 (53.9%)	
	Regional	1717 (17.9%)	2583 (26.5%)	230 (19.9%)	948 (20.8%)	888 (20.7%)	6366 (21.7%)	
	Distant	424 (4.4%)	4819 (49.5%)	489 (42.2%)	261 (5.7%)	241 (5.6%)	6234 (21.2%)	
	Unknown	130 (1.4%)	406 (4.2%)	100 (8.6%)	152 (3.3%)	132 (3.1%)	920 (3.1%)	
Grade								<0.001
	Low	4390 (45.7%)	1587 (16.3%)	167 (14.4%)	1654 (36.2%)	1758 (41.0%)	9556 (32.6%)	
	High	2228 (23.2%)	2534 (26.0%)	249 (21.5%)	569 (12.5%)	642 (15.0%)	6222 (21.2%)	
	Other^3^	29 (0.3%)	0 (0%)	51 (4.4%)	8 (0.2%)	19 (0.4%)	107 (0.4%)	
	Unknown	2955 (30.8%)	5614 (57.7%)	691 (59.7%)	2332 (51.1%)	1872 (43.6%)	13,464 (45.9%)	
Surgery								<0.001
	Yes	9036 (94.1%)	6140 (63.1%)	681 (58.8%)	4188 (91.8%)	3903 (91.0%)	23,948 (81.6%)	
	No	546 (5.7%)	3398 (34.9%)	456 (39.4%)	347 (7.6%)	367 (8.6%)	5114 (17.4%)	
	Unknown	20 (0.2%)	197 (2.0%)	21 (1.8%)	28 (0.6%)	21 (0.5%)	287 (1.0%)	
Chemotherapy								<0.001
	No/unknown	9306 (96.9%)	8213 (84.4%)	982 (84.8%)	4492 (98.4%)	4211 (98.1%)	27,204 (92.7%)	
	Yes	296 (3.1%)	1522 (15.6%)	176 (15.2%)	71 (1.6%)	80 (1.9%)	2145 (7.3%)	
Radiotherapy								<0.001
	No/unknown	9476 (98.7%)	7939 (81.6%)	990 (85.5%)	4470 (98.0%)	4213 (98.2%)	27,088 (92.3%)	
	Yes	126 (1.3%)	1796 (18.4%)	168 (14.5%)	93 (2.0%)	78 (1.8%)	2261 (7.7%)	

^1^ Other includes American Indian/Alaska Native and Asian/Pacific Islander. ^2^ The time interval is set as the lower limit. ^3^ Other includes B-cell, pre-B, B-precursor and T-cell. CVD, cardiovascular disease; SEER, Surveillance, Epidemiology, and End Results.

### 3.2 Distribution of Deaths

**Fig. 1.**
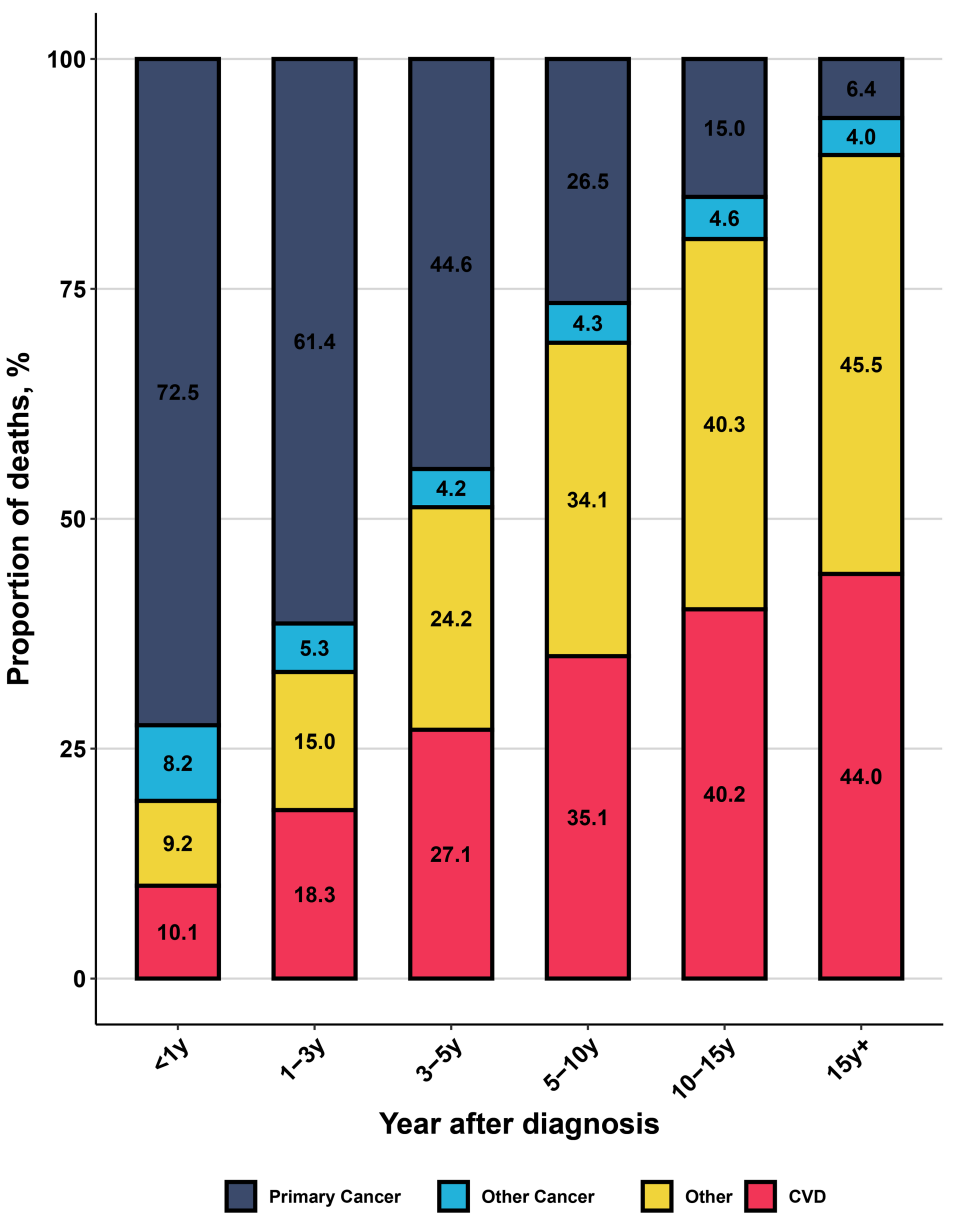
**The distribution of all causes of death in older kidney cancer patients**. y, year; CVD, cardiovascular disease.

**Fig. 2.**
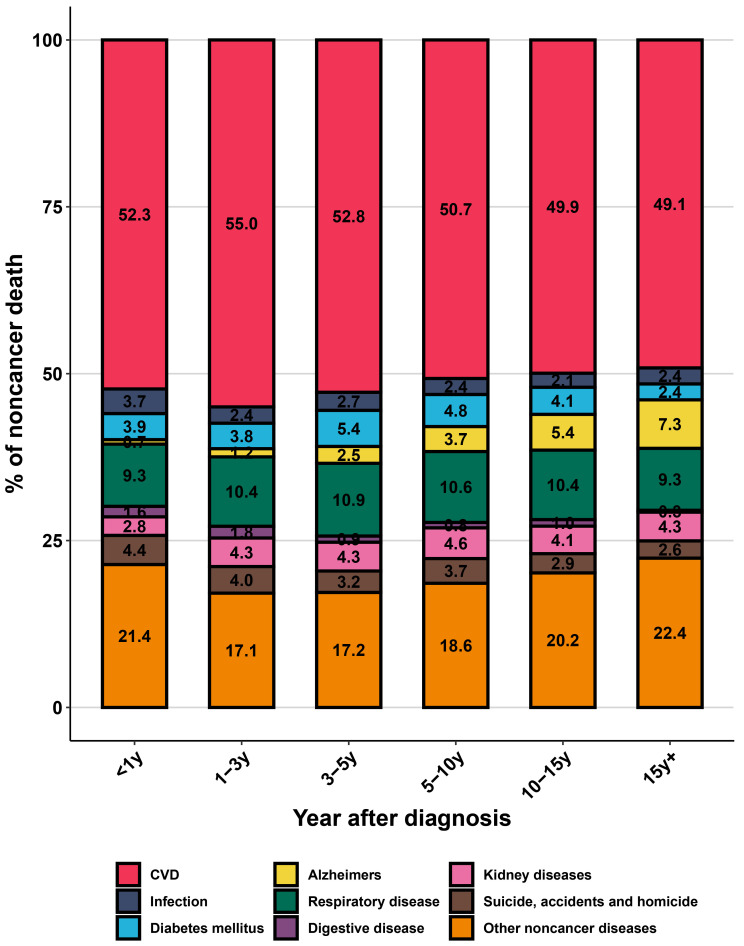
**The distribution of non-cancer death in older kidney cancer patients**. y, year; CVD, cardiovascular disease.

Older kidney cancer patients are more prone to mortality from non-cancer causes, with CVD being a dominant non-cancer cause of death. As survival time lengthened, the proportion of primary cancer death decreased steadily (from 72.5% to 6.4%), while the proportion of CVD death increased significantly (from 10.1% to a peak of 44.0%), surpassing primary cancer death at 5–10 years after diagnosis (Fig. 1). Among all non-cancer deaths, CVD death was the leading cause, followed by other non-cancer diseases and respiratory diseases (Fig. 2). In subgroup analysis, the proportion of CVD death in older patients with localized kidney cancer was much higher than primary cancer death (37.7% vs. 22.7%) (**Supplementary Fig. 2**). Similarly, older patients with low-grade kidney cancer exhibited an elevated proportion of CVD death than those with high-grade kidney cancer. Details on the proportions of CVD and other competing causes of death in various subgroups can be found in **Supplementary Fig. 2**.

### 3.3 Cumulative Mortality

**Fig. 3.**
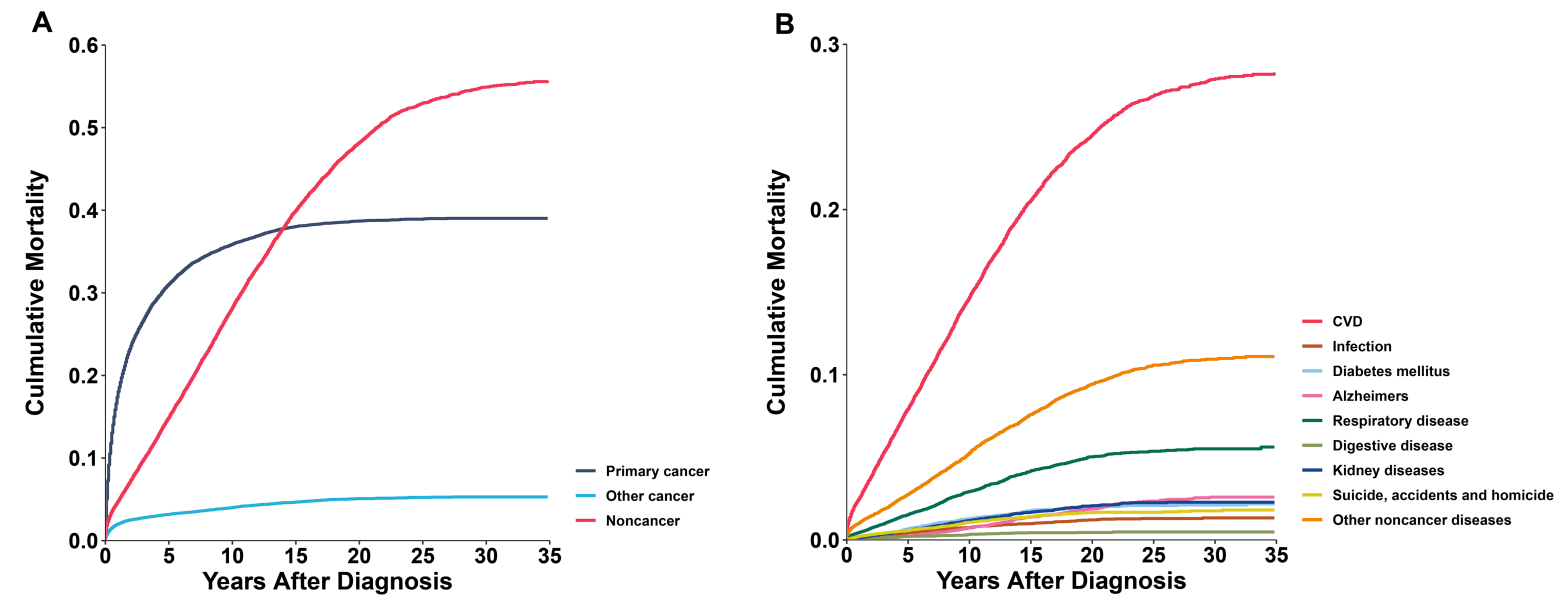
**Cumulative mortality in older kidney cancer patients**. (A) All causes of death. (B) Non-cancer death. CVD, cardiovascular disease.

In older kidney cancer patients, the cumulative mortality of primary cancer rose rapidly within 5 years after diagnosis, followed by a slower increase. Between 10 and 15 years after diagnosis, deaths from non-cancer causes surpassed that from the primary cancer (Fig. 3A). Analyzing the cumulative mortality rates of non-cancer causes, CVD exhibited a significantly higher cumulative mortality rate than other diseases and continued to rise with increasing survival time (Fig. 3B). In subgroup analysis, the phenomenon of non-cancer cumulative mortality exceeding primary cancer also occurred in all genders, white and black populations, localized and low-grade tumors, and patients who underwent surgery (**Supplementary Fig. 3**).

### 3.4 Mortality Compared to the General Population

**Fig. 4.**
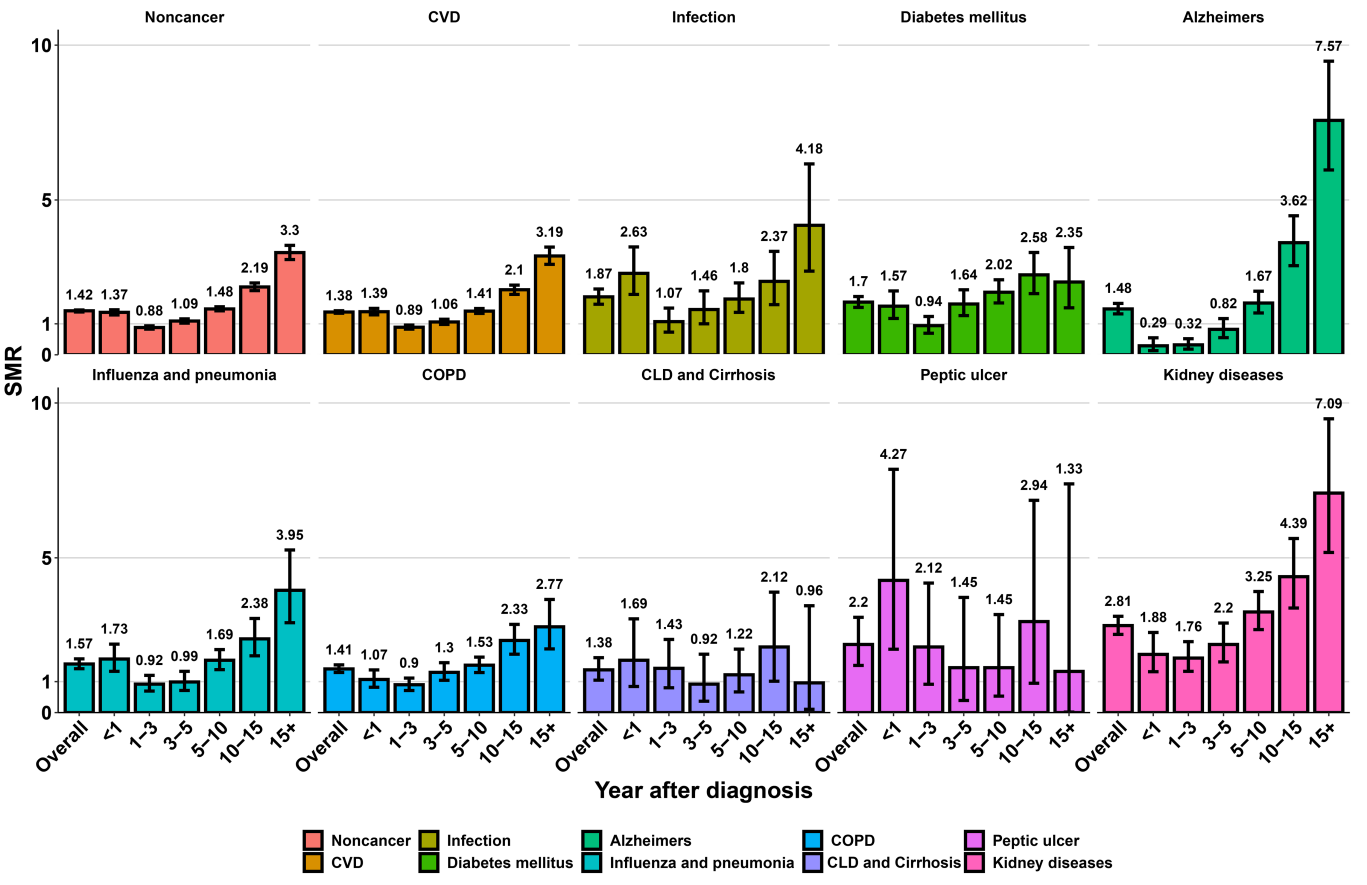
**Standardized mortality ratios for each non-cancer cause of death in older kidney cancer patients**. SMR, standardized mortality ratio; CVD, cardiovascular disease; COPD, chronic obstructive pulmonary disease; CLD, chronic liver disease.

Overall, older kidney cancer patients faced an elevated risk of CVD and other non-cancer death in comparison with the general older population (SMR: 1.38–2.81; AER: 1.1–143.69) (Fig. 4 and **Supplementary Table 2**). The risk of CVD mortality was elevated within the first year after diagnosis (SMR: 1.39, 95% CI: 1.29–1.50; AER: 88.45), with a prominent drop 1–3 years after diagnosis (SMR: 0.89, 95% CI: 0.83–0.96; AER: –24.49), and then gradually rose. After 5 years post-diagnosis, the CVD death risk in older kidney cancer patients was once again higher than that of the general older population and continued to increase with survival time (SMR: 1.41–3.19; AER: 93.38–496.21). In analyses of SMRs for other competing causes of death, with the exception of diabetes mellitus, chronic liver disease (CLD) and cirrhosis, and peptic ulcer, there was also an increased trend in SMRs with increasing survival time after 5 years post-diagnosis among older kidney cancer patients. In comparison to the expected death in general older population, older kidney cancer patients displayed higher SMRs and AERs for CVD mortality across different clinical characteristics, regardless of the sex, race, years of diagnosis, SEER stage, and surgery (**Supplementary Table 3**).

## 4. Discussion

To our knowledge, this research represents the first large-scale, long-term follow-up investigation comprehensively assessing the risk of CVD and other competing causes of death among 29,349 older kidney cancer patients. In this population-based study, we observed a progressive rise in the proportion of non-cancer deaths with prolonged survival time, with CVD death emerging as the predominant non-cancer death in this demographic. Older kidney cancer patients exhibited a heightened susceptibility to CVD death compared to the general older population.

Primary cancer, CVD, and other non-cancer deaths were the primary causes of death for the entire cohort of older kidney cancer patients. The proportions of CVD death and other non-cancer deaths escalated over the years following diagnosis, with CVD mortality surpassing primary cancer mortality between 5 to 10 years after diagnosis. These findings are consistent with the research of Zaorsky *et al*. [8], indicating that non-cancer deaths predominate over index or non-index cancer deaths in patients with kidney and renal pelvis cancer, with heart disease emerging as the most prevalent non-cancer cause of death. Similarly, a population-based study revealed a progressive increase in the proportion of CVD death among stage I/II renal cell carcinoma patients over time, surpassing primary cancer mortality between 5 to 10 years after diagnosis, albeit without stratification by older age subgroups [12]. Employing competing risk models, we further evaluated the risk of CVD and other competing deaths by calculating cumulative mortality, ensuring the precision of our results. The cumulative mortality for CVD markedly exceeded that of other non-cancer conditions. This aligns with broader findings suggesting that for patients aged 65 and above at initial cancer diagnosis, the cumulative incidence of death attributable to CVD surpasses that of other competing events [28]. Consequently, CVD assumes primacy in the prevention and treatment of non-cancer comorbidities for older kidney cancer patients.

Despite the generally higher CVD risk among older patients compared to younger cohorts, the risk of CVD mortality remains elevated among older kidney cancer patients within the first year and 5 years post-diagnosis, relative to the general older population. Studies on cancers such as gallbladder, esophageal, and gastric cancers have similarly indicated an initial rise followed by a subsequent decline in CVD risk within the first year after diagnosis [29, 30, 31]. Research on survivors of T1N0M0 renal cell carcinoma supports our findings, demonstrating a modest risk of death due to heart disease within 1–5 years after diagnosis, but a significantly elevated risk >5 years after diagnosis [18]. Surprisingly, older kidney cancer patients exhibit lower CVD mortality risks than the general older population 1-3 years after diagnosis, with no discernible elevation in CVD mortality risk 3–5 years after diagnosis. Plausible explanations include heightened health consciousness and regular health monitoring after diagnosis in older kidney cancer patients, coupled with healthier behaviors and lifestyles adopted upon diagnosis with kidney cancer, such as smoking cessation leading to a halving of the elevated coronary heart disease risk after just one year [32]. The higher risk of CVD death more than five years after diagnosis is due to longer exposure to cardiovascular risk factors with longer survival in older patients [33, 34].

The heightened risk of CVD mortality among older kidney cancer patients stems from multifactorial factors. Firstly, older cancer patients typically harbor more cardiovascular risk factors and comorbidities, including hypertension, diabetes, and coronary artery disease, alongside a history of being overweight and smoking [35, 36], which could increase their risk of CVD death. Secondly, the complex pathophysiology of cardiorenal syndrome, such as atherosclerosis, hypertension, heart failure, and chronic inflammation, accelerates CVD progression [37]. Additionally, complex interrelations exist between aging, cancer, and CVD, with older cancer patients experiencing an elevated CVD risk due to intersecting biological mechanisms such as inflammation, cellular senescence, and telomere attrition [38]. Furthermore, over the past decade, tyrosine kinase inhibitors (e.g., sunitinib) have been utilized as first-line treatments for metastatic kidney cancer patients, markedly enhancing survival rates [39, 40]. However, the attendant cardiovascular toxicities cannot be overlooked [41, 42, 43]. Lastly, CVD risk might significantly increase after kidney cancer surgery [44, 45, 46, 47]. The issue of excessive surgeries for kidney masses may further compound the risk of CVD mortality among older kidney cancer patients [48]. Concurrently, psychological burden associated with cancer diagnosis and treatment may precipitate additional psychological stress, potentially leading to cardiovascular events [49].

We investigated the causes of death across different subgroups of older kidney cancer patients. Notably, the SMR for CVD among older kidney cancer patients decreased with increasing year of diagnosis, indicating a lower CVD risk among older kidney cancer patients in later years compared to matched general population. Similar trends have been observed in prostate cancer research [49]. These shifts may be attributed to advancements in cardio-oncology, facilitating improvements in the management and prevention of CVD among older kidney cancer patients [10, 13, 34, 50, 51]. Surgery remains the major treatment in kidney cancer guidelines [52, 53]. Due to the finding of accelerated kidney insufficiency and adverse cardiovascular effects of radical nephrectomy, there has been a major shift in surgical treatment of kidney cancer from radical nephrectomy to partial nephrectomy [44, 54], which has favored the reduction of the risk of death from CVD in older kidney cancer patients. Research on prognostic models for older kidney cancer patients is also ongoing [55]. These will help to alleviate cardiovascular toxicity and adverse consequences for older kidney cancer patients, and mitigate the risk of CVD mortality. Our findings corroborate previous research indicating a heightened CVD risk among localized, low-grade kidney cancer patients, likely attributable to their prolonged survival duration, thereby increasing their susceptibility to CVD, given its usually chronic nature [33]. However, staged, older kidney cancer patients exhibit a higher risk of CVD death compared to the general population, despite the majority of distant older kidney cancer patients primarily succumbing to primary cancer and other cancers. Yu *et al*. [12] similarly found the risk of CVD death was elevated across all stages of patients with kidney cancer compared to the general population.

Strengths of our study lie in its extensive multicenter design and prolonged follow-up period. Among investigations evaluating risk of CVD and other competing mortality causes in older kidney cancer patients, our study ranks as one of the largest. The large sample size allowed for thorough analyses of CVD and other competing mortality causes, considering various patient characteristics. The prolonged follow-up period facilitated the evaluation of risks of CVD and other competing causes of death, both short-term and long-term.

However, our study has some limitations. Firstly, treatment modalities have evolved over the past 40 years and different types of surgery and treatment doses may influence the causes of death among older kidney cancer patients. However, detailed treatment information is lacking in the SEER Program, precluding exploration of this aspect. Secondly, the database does not include data on patient comorbidities and health status, impeding analysis of the impact of comorbidities and other risk factors on different causes of death. Thirdly, the present study was based only on the SEER database, which may cause some bias, and external validation using another independent dataset should be performed in the future to improve the credibility of the study. Finally, the SEER database may contain potential misclassifications of death causes due to biases in death certificate reporting. Nevertheless, the SEER Program ensures data accuracy through systematic and standardized procedures.

## 5. Conclusions

In conclusion, our study findings indicate that as survival time prolongs, older kidney cancer patients are more probably to succumb to non-cancer causes than from the kidney cancer, with CVD emerging as the primary non-cancer cause of death among this demographic. Older kidney cancer patients face an increased risk of CVD death compared to the general population. Our study results underscore the importance of preventing and managing non-cancer death among older kidney cancer patients, particularly CVD death.

## Data Availability

The datasets analyzed in this study are publicly available from the SEER database (https://seer.cancer.gov).

## Abbreviations

AERs, absolute excess risks; CDC, Centers for Disease Control and Prevention; CLD, chronic liver disease; CVD, cardiovascular disease; ICD-10, International Classification of Diseases, 10th Revision; SMRs, standardized mortality ratios; SEER, Surveillance, Epidemiology, and End Results; WONDER, Wide-ranging Online Data for Epidemiologic Research; y, year.

## Supplementary Material

Supplementary material associated with this article can be found, in the online version, at https://doi.org/10.31083/RCM25277.
