# Supplementary material for: Cardiovascular Disease and Other Competing Causes of Death in Older Kidney Cancer Patients
Source: Rev Cardiovasc Med. 2025 Jan 14;26(1):25277. doi: 10.31083/RCM25277 (PMC11759974; doi:10.31083/RCM25277)
Supplement: Supplementary file 1 [file 2153-8174-26-1-25277-s1.docx]

***Supplementary Material***

**Cardiovascular disease and Other Competing Causes of Death in Older Kidney Cancer Patients**

**List of Supplementary Tables**

Supplementary Table 1. The classifications of non-cancer cause of death.

Supplementary Table 2. SMRs and AERs for each non-cancer cause of deaths in older kidney cancer patients by year after diagnosis.

Supplementary Table 3. SMRs and AERs of CVD in older kidney cancer patients by clinical characteristic.

**List of Supplementary Figures**

Supplementary Fig. 1 Flow diagram of cohort selection criteria.

Supplementary Fig. 2 The distribution of all causes of death in older kidney cancer patients by clinical characteristic.

Supplementary Fig. 3 Cumulative mortality in older kidney cancer patients by clinical characteristic.

**Supplementary Table 1. The classifications of non-cancer cause of death.**

| **Non-cancer cause of death** | **Cardiocerebrovascular diseases** | **Infection** | **Diabetes Mellitus** | **Alzheimer's disease** | **Respiratory diseases** | **Digestive diseases** | **Kidney diseases** | **Suicide, accidents and homicide** | **Other non-neoplastic diseases** |
| --- | --- | --- | --- | --- | --- | --- | --- | --- | --- |
| **Specific classifications** | Aortic Aneurysm and Dissection  I71 | Other Infectious and Parasitic Diseases including  HIV  A00–B99 | Diabetes Mellitus  E10–E14 | Alzheimer's (ICD-9 and 10 only)  G30 | Chronic Obstructive Pulmonary Disease and Allied Cond  J44.8–J44.9 | Chronic Liver Disease and Cirrhosis  K76.9 K74 | Nephritis, Nephrotic Syndrome and Nephrosis  N03–N05 | Accidents and Adverse Effects  V01–X59 | Certain Conditions Originating in Perinatal Period  P70.3-P74 |
| **Specific classifications** | Atherosclerosis  I70 | Septicemia  A41 |  |  | Pneumonia and Influenza  J09–J18 | Stomach and Duodenal Ulcers  B00.0–B00.2, B00.7–B00.8 |  | Homicide and Legal Intervention  X60–X84 | Complications of Pregnancy, Childbirth, Puerperium  O94 |
|  | Cerebrovascular Diseases  I60–I69 | Tuberculosis  A15–A19 |  |  |  |  |  | Suicide and Self-Inflicted Injury  X60–X84 | Congenital Anomalies  Q00–Q99 |
|  | Diseases of Heart  I00-I09  I11 I13  I20-I51 | Syphilis  A50-A52 |  |  |  |  |  |  | Other Cause of Death  O80–O84 |
|  | Hypertension without Heart Disease  I10 I12 |  |  |  |  |  |  |  | Symptoms, Signs and Ill-Defined Conditions  R00–R57.1, R57.8–R59.9, R65.2–R65.3,  R68.0–R94, R96–R99 |
|  | Other Diseases of Arteries, Arterioles, Capillaries  I72-I78 |  |  |  |  |  |  |  |  |

**Supplementary Table 2. SMRs and AERs for each non-cancer cause of deaths in older kidney cancer patients by year after diagnosis.**

| **Year after diagnosis** | **Cause of death** | **SMR (95%CI)** | **AER** |
| --- | --- | --- | --- |
| Overall | Noncancer | 1.42(1.38-1.45) | 143.69 |
| <1 | Noncancer | 1.37(1.29-1.45) | 127.23 |
| 1-3 | Noncancer | 0.88(0.83-0.93) | -41.55 |
| 3-5 | Noncancer | 1.09(1.02-1.16) | 30.86 |
| 5-10 | Noncancer | 1.48(1.42-1.55) | 167.35 |
| 10-15 | Noncancer | 2.19(2.07-2.32) | 413.17 |
| 15+ | Noncancer | 3.3(3.08-3.53) | 795.61 |
| Overall | CVD | 1.38(1.34-1.42) | 86.43 |
| <1 | CVD | 1.39(1.29-1.50) | 88.45 |
| 1-3 | CVD | 0.89(0.83-0.96) | -24.49 |
| 3-5 | CVD | 1.06(0.98-1.15) | 13.91 |
| 5-10 | CVD | 1.41(1.34-1.49) | 93.38 |
| 10-15 | CVD | 2.1(1.95-2.25) | 248.36 |
| 15+ | CVD | 3.19(2.92-3.48) | 496.21 |
| Overall | Infection | 1.87(1.63-2.12) | 7.29 |
| <1 | Infection | 2.63(1.95-3.48) | 13.76 |
| 1-3 | Infection | 1.07(0.73-1.51) | 0.57 |
| 3-5 | Infection | 1.46(1.00-2.07) | 3.89 |
| 5-10 | Infection | 1.8(1.37-2.32) | 6.73 |
| 10-15 | Infection | 2.37(1.62-3.34) | 11.50 |
| 15+ | Infection | 4.18(2.70-6.16) | 26.74 |
| Overall | Diabetes mellitus | 1.7(1.53-1.88) | 10.47 |
| <1 | Diabetes mellitus | 1.57(1.17-2.06) | 8.56 |
| 1-3 | Diabetes mellitus | 0.94(0.70-1.24) | -0.93 |
| 3-5 | Diabetes mellitus | 1.64(1.27-2.10) | 9.65 |
| 5-10 | Diabetes mellitus | 2.02(1.67-2.42) | 15.32 |
| 10-15 | Diabetes mellitus | 2.58(1.98-3.30) | 23.61 |
| 15+ | Diabetes mellitus | 2.35(1.52-3.47) | 20.18 |
| Overall | Alzheimers | 1.48(1.32-1.66) | 6.81 |
| <1 | Alzheimers | 0.29(0.13-0.55) | -10.04 |
| 1-3 | Alzheimers | 0.32(0.18-0.52) | -9.61 |
| 3-5 | Alzheimers | 0.82(0.55-1.17) | -2.57 |
| 5-10 | Alzheimers | 1.67(1.35-2.05) | 9.52 |
| 10-15 | Alzheimers | 3.62(2.88-4.49) | 36.93 |
| 15+ | Alzheimers | 7.57(5.97-9.48) | 92.76 |
| Overall | Influenza and pneumonia | 1.57(1.42-1.74) | 9.54 |
| <1 | Influenza and pneumonia | 1.73(1.33-2.21) | 12.23 |
| 1-3 | Influenza and pneumonia | 0.92(0.70-1.20) | -1.28 |
| 3-5 | Influenza and pneumonia | 0.99(0.72-1.33) | -0.19 |
| 5-10 | Influenza and pneumonia | 1.69(1.39-2.03) | 11.51 |
| 10-15 | Influenza and pneumonia | 2.38(1.83-3.04) | 23.10 |
| 15+ | Influenza and pneumonia | 3.95(2.90-5.25) | 49.36 |
| Overall | COPD | 1.41(1.30-1.54) | 10.51 |
| <1 | COPD | 1.07(0.82-1.38) | 1.79 |
| 1-3 | COPD | 0.9(0.71-1.12) | -2.60 |
| 3-5 | COPD | 1.3(1.04-1.61) | 7.72 |
| 5-10 | COPD | 1.53(1.29-1.79) | 13.40 |
| 10-15 | COPD | 2.33(1.89-2.85) | 33.76 |
| 15+ | COPD | 2.77(2.06-3.65) | 44.94 |
| Overall | CLD and Cirrhosis | 1.38(1.05-1.77) | 1.10 |
| <1 | CLD and Cirrhosis | 1.69(0.84-3.03) | 2.04 |
| 1-3 | CLD and Cirrhosis | 1.43(0.80-2.36) | 1.27 |
| 3-5 | CLD and Cirrhosis | 0.92(0.37-1.89) | -0.25 |
| 5-10 | CLD and Cirrhosis | 1.22(0.67-2.05) | 0.65 |
| 10-15 | CLD and Cirrhosis | 2.12(1.01-3.89) | 3.28 |
| 15+ | CLD and Cirrhosis | 0.96(0.11-3.45) | -0.13 |
| Overall | Peptic ulcer | 2.2(1.52-3.08) | 1.27 |
| <1 | Peptic ulcer | 4.27(2.05-7.86) | 3.47 |
| 1-3 | Peptic ulcer | 2.12(0.91-4.18) | 1.19 |
| 3-5 | Peptic ulcer | 1.45(0.39-3.72) | 0.48 |
| 5-10 | Peptic ulcer | 1.45(0.53-3.17) | 0.48 |
| 10-15 | Peptic ulcer | 2.94(0.95-6.86) | 2.05 |
| 15+ | Peptic ulcer | 1.33(0.02-7.39) | 0.35 |
| Overall | Kidney diseases | 2.81(2.53-3.11) | 16.11 |
| <1 | Kidney diseases | 1.88(1.32-2.59) | 7.82 |
| 1-3 | Kidney diseases | 1.76(1.33-2.29) | 6.82 |
| 3-5 | Kidney diseases | 2.2(1.64-2.89) | 10.70 |
| 5-10 | Kidney diseases | 3.25(2.68-3.91) | 20.09 |
| 10-15 | Kidney diseases | 4.39(3.38-5.62) | 30.29 |
| 15+ | Kidney diseases | 7.09(5.17-9.49) | 54.36 |

SMR, standard mortality ratio; AER, absolute excess risk; CVD, cardiovascular disease; COPD, chronic obstructive pulmonary disease; CLD, chronic liver disease.

**Supplementary Table 3. SMRs and AERs of CVD in older kidney cancer patients by clinical characteristic.**

| **Characteristic** | **SMR (95%CI)** | **AER** |
| --- | --- | --- |
| **Gender** |  |  |
| Male | 1.38(1.33-1.44) | 90.48 |
| Female | 1.25(1.20-1.31) | 59.11 |
| **Race** |  |  |
| White | 1.38(1.34-1.43) | 86.70 |
| Black | 1.45(1.31-1.60) | 113.71 |
| Other^1^ | 1.95(1.71-2.22) | 115.84 |
| **Year of diagnosis** |  |  |
| 1975-1983 | 2.46(2.28-2.64) | 329.70 |
| 1984-1993 | 2.07(1.96-2.18) | 241.86 |
| 1994-2003 | 1.46(1.39-1.54) | 104.91 |
| 2004-2018 | 0.80(0.76-0.85) | -45.27 |
| **SEER stage** |  |  |
| Localized | 1.33(1.29-1.38) | 75.52 |
| Regional | 1.41(1.32-1.51) | 93.58 |
| Distant | 1.57(1.38-1.77) | 128.04 |
| **Grade** |  |  |
| Low | 1.16(1.11-1.22) | 37.20 |
| High | 0.98(0.90-1.06) | -4.72 |
| **Surgery** |  |  |
| Yes | 2.48(2.22-2.75) | 334.82 |
| No | 1.32(1.28-1.36) | 72.89 |
| **Radiotherapy** |  |  |
| No | 1.38(1.34-1.42) | 85.81 |
| Yes | 1.24(1.00-1.52) | 53.67 |
| **Chemotherapy** |  |  |
| No | 1.39(1.35-1.43) | 88.13 |
| Yes | 0.85(0.67-1.08) | -33.19 |

^1^ Other includes American Indian/Alaska Native and Asian/Pacific Islander. SMR, standard mortality ratio; AER, absolute excess risk.


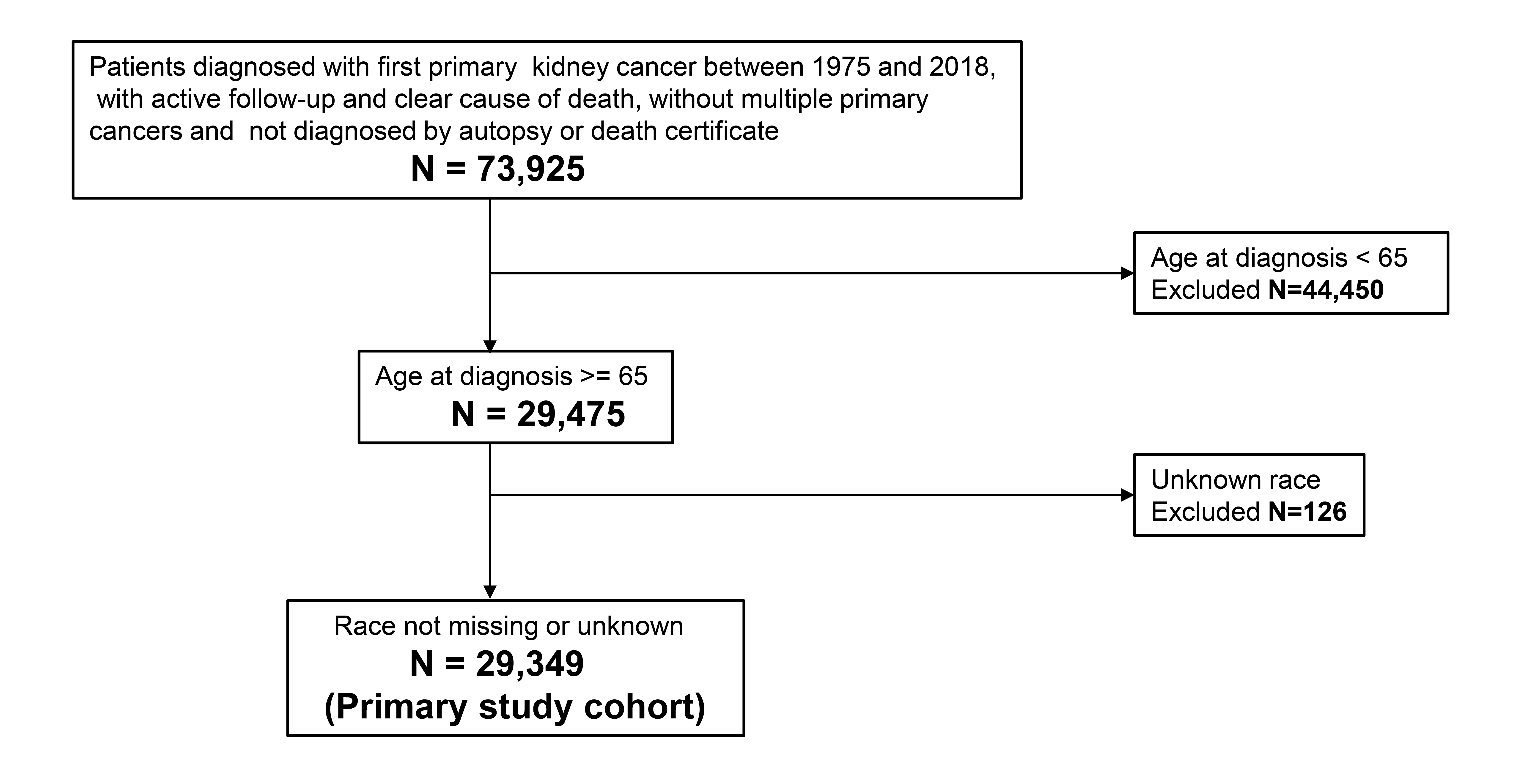


**Supplementary Fig. 1 Flow diagram of cohort selection criteria.**


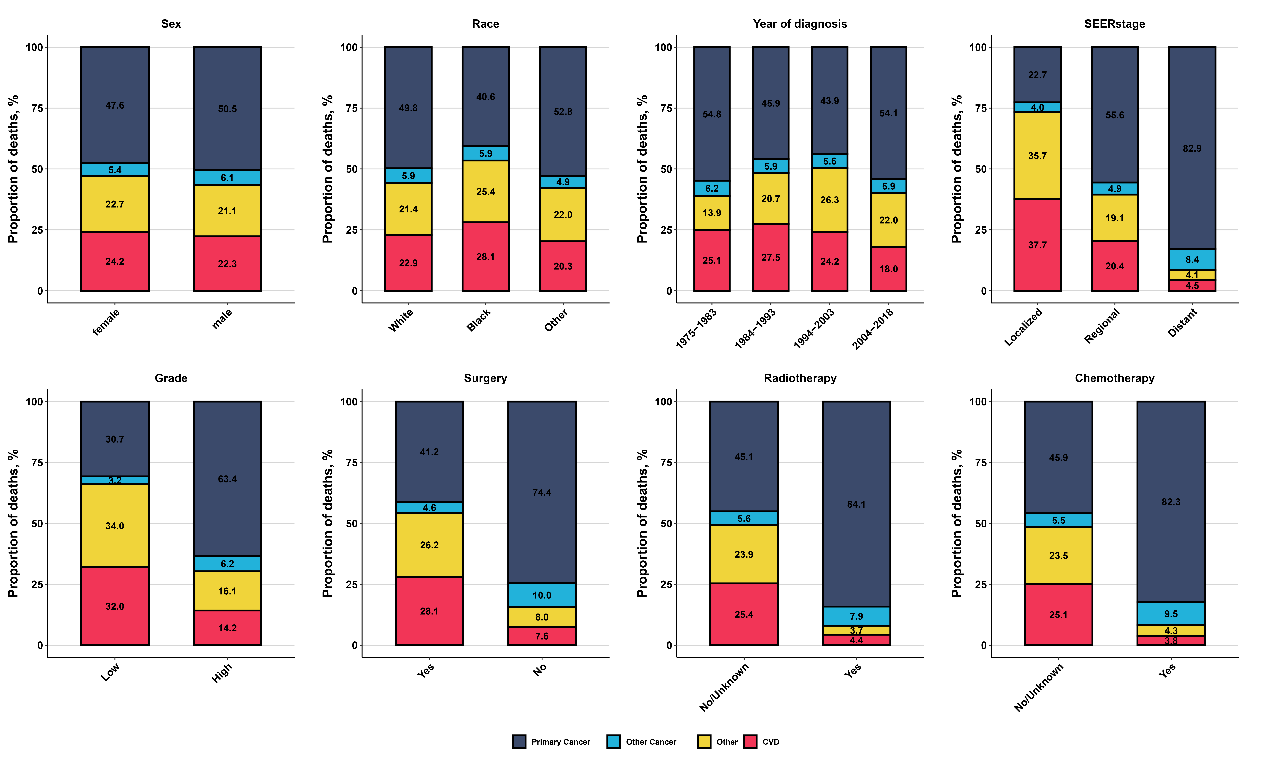


**Supplementary Fig. 2 The distribution of all causes of death in older kidney cancer patients by clinical characteristic.**


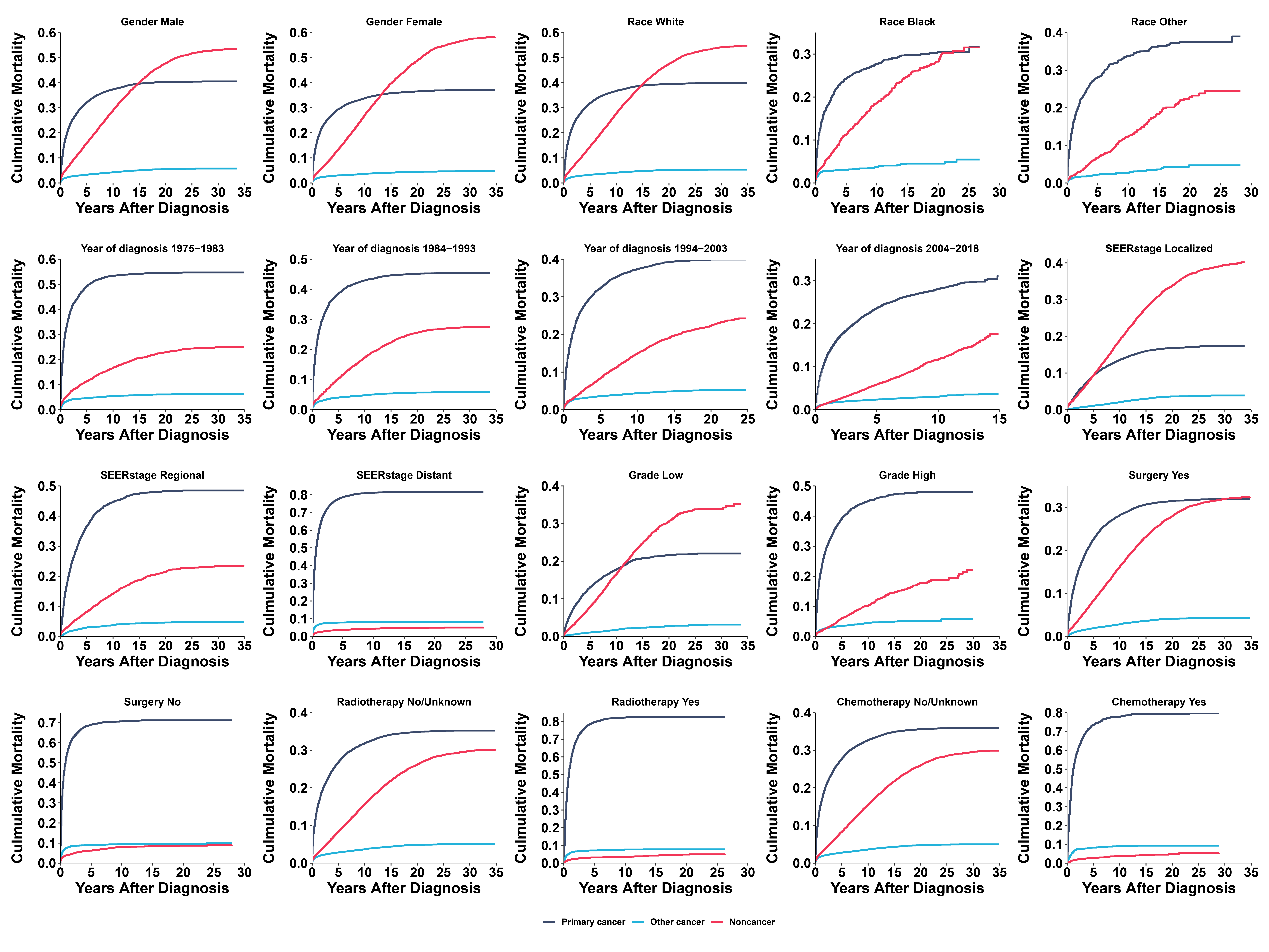


**Supplementary Fig. 3 Cumulative mortality in older kidney cancer patients by clinical characteristic.**
